# LINC00702 suppresses proliferation and invasion in non-small cell lung cancer through regulating miR-510/PTEN axis

**DOI:** 10.18632/aging.101846

**Published:** 2019-03-06

**Authors:** Wencheng Yu, Daowei Li, Xiaoyan Ding, Yong Sun, Yanli Liu, Jinpeng Cong, Jiong Yang, Jian Sun, Xuchao Ning, Hongmei Wang, Tao Xu

**Affiliations:** 1 Department of Respiratory, the Affiliated Hospital of Qingdao University, Qingdao, Shandong 266003, China; 2 Department of Respiratory, Shandong Provincial Hospital Affiliated to Shandong University, Jinan, Shandong 250021, China; 3 Department of Pathology, the Affiliated Hospital of Qingdao University, Qingdao, Shandong 266003, China; 4 Department of Geriatrics, the Affiliated Hospital of Qingdao University, Qingdao, Shandong 266003, China; * Equal contribution

**Keywords:** non-small cell lung cancer, LINC00702, miR-510, PTEN

## Abstract

Background: Long non-coding RNAs (lncRNAs) have been consistently reported to be involved in the progression of non-small cell lung cancer (NSCLC). In this study, we aimed to identify aberrantly expressed lncRNAs in NSCLC, in order to explore new therapeutic targets for NSCLC.

Methods: Two pairs of NSCLC and adjacent normal tissues were first analyzed by RNA sequencing. The expressions of LINC00702 in 40 pairs patient samples and in 4 NSCLC cell lines was measured by quantitative real-time PCR. Putative target miRNAs of LINC00702 were predicted by the bioinformatics tools. The effect of LINC00702 on tumor growth *in vivo* was evaluated.

Results: LINC00702 was significantly down-regulated in patients with NSCLC, which was correlated with tumor size and metastasis. In addition, overexpression of LINC00702 markedly suppressed proliferation and metastasis in NSCLC cells via inducing apoptosis *in vitro* and *in vivo*. Moreover, bioinformatics and luciferase reporter assays demonstrated that LINC00702 functioned as a competing endogenous RNA (ceRNA) for miR-510 in NSCLC, and upregulated its target gene PTEN.

Conclusion: Our results indicated that LINC00702 modulated the expression of PTEN gene by acting as a ceRNA for miR-510 in NSCLC. Therefore, LINC00702 may serve as a potential target for the diagnosis and treatment of patients with NSCLC.

## Introduction

Lung cancer is the leading cause of cancer-related mortality worldwide. Non-small cell lung cancer (NSCLC) accounts for about 80% of all lung cancer cases and about 75% of the patients are diagnosed in the advanced stages of the disease, with a 5-year overall survival rate < 15% [1]. Although many research groups are actively involved in studying the pathogenesis of NSCLC, the underlying mechanisms have not been fully elucidated and the efficacy of the current therapies is not satisfactory [1]. Therefore, in order to identify new therapeutic targets and improve clinical outcome of patients, the molecular basis for NSCLC needs to be elucidated.

Long non-coding RNAs (lncRNAs) are a group of non-protein coding RNAs longer than 200 nucleotides [2]. In recent years, studies have found that several lncRNAs are abnormally expressed in lung cancer tissues, which are associated with tumor invasion, metastasis and prognosis [2,3]. Since lncRNAs play important roles in tumorigenesis, they are increasingly been considered as novel targets for tumor therapy [2].

MicroRNAs (miRNAs) are single-stranded small non-coding RNAs about 19 to 25 nucleotides in length, which regulate gene expression through translational repression [4]. In theory, any RNA species can bind to miRNA and thus act as a competitive endogenous RNA (ceRNA). MiRNAs play a central role in the ceRNA regulatory network and repress gene expression by binding to target mRNAs [5]. Recent studies indicated that miRNA-lncRNA interactions plays an important role in human complex diseases [6,7]. The theory of ceRNA could explain this interaction mechanism [8]. In this theory, LncRNA can consider as a ceRNA, which binding some miRNAs competitively by base complementation. In that case, the binding probability between miRNAs and their target genes were reduced immensely, leading to some changes in level of the target genes of miRNAs indirectly [9].

In this study, to identify aberrantly expressed lncRNAs in NSCLC, two pairs of NSCLC and their adjacent normal tissues were first analyzed by RNA-Seq. We found LINC00702 was significantly downregulated in tumor tissues, which correlated with the tumor growth and metastasis of patients with NSCLC. Thus, we aimed to investigate the mechanisms by which LINC00702 regulated the tumorgenesis of NSCLC.

## RESULTS

### LINC00702 was downregulated in patients with NSCLC

**Figure 1:**
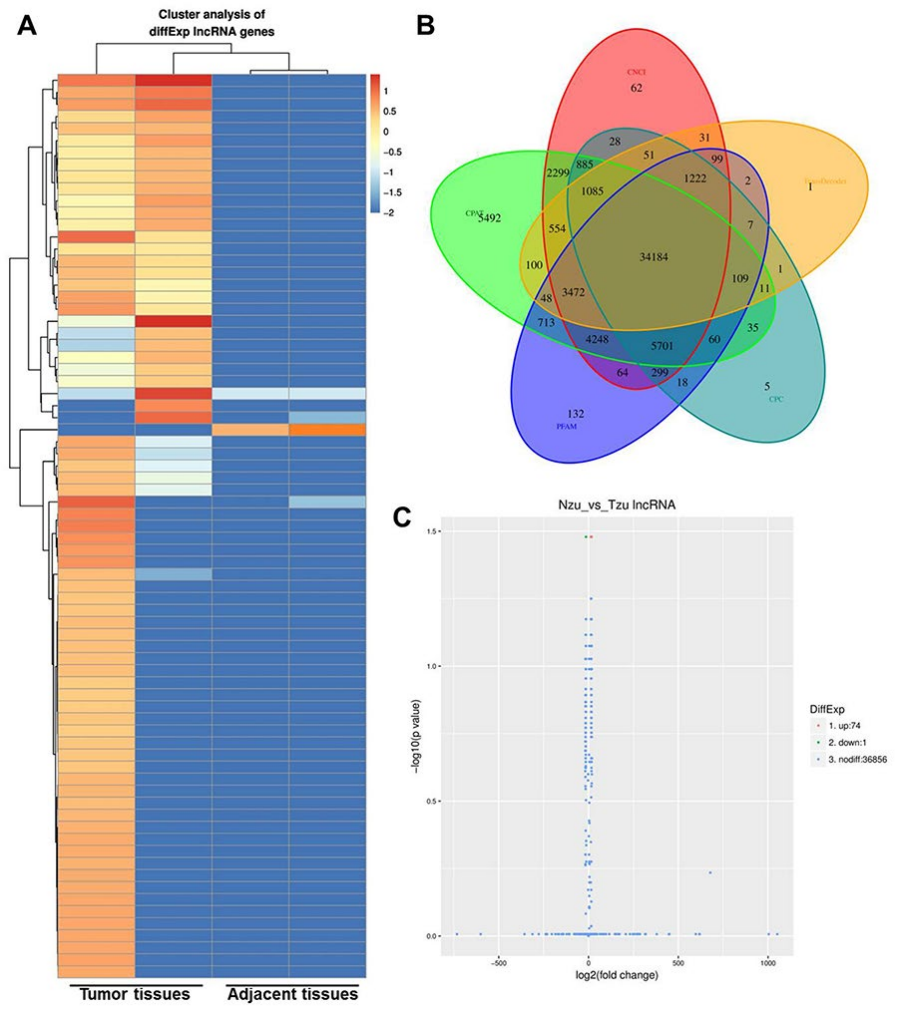
**Identification of the aberrant expression of lncRNA in the NSCLC tissues.** (**A**) Heatmap of lncRNA expression levels (log2RPKM) in two NSCLC tissue and adjacent normal tissues. (**B**) Code potential screening Venn diagram. (**C**) LncRNA differentially expressed volcano plots.

To determine the correlation between aberrant expression of lncRNAs and NSCLC, two pairs of tumor and adjacent normal tissues were initially analyzed by RNA-seq. Differential expressed genes were analyzed using clustering analysis which were marked in red (Figure. 1A, 1B). 74 upregulated lncRNAs and 1 downregulated lncRNAs were observed between NSCLC and adjacent tissues (Figure 1C). Meanwhile, DEGs between NSCLC and adjacent tissues were recorded in Volcano plot with the criteria of FDR < 0.01 or log2 fold-change (FC) ≥ 4, which were marked in yellow (Figure. 1C). Among these DEGs, LINC00702 was picked up with a great significance of which log2FC was -4.5926. LINC00702 was markedly reduced in NSCLC according to the data. Thus, we focused on investigating the role of LINC00702 during the tumorgenesis in NSCLC.

**Figure 2:**
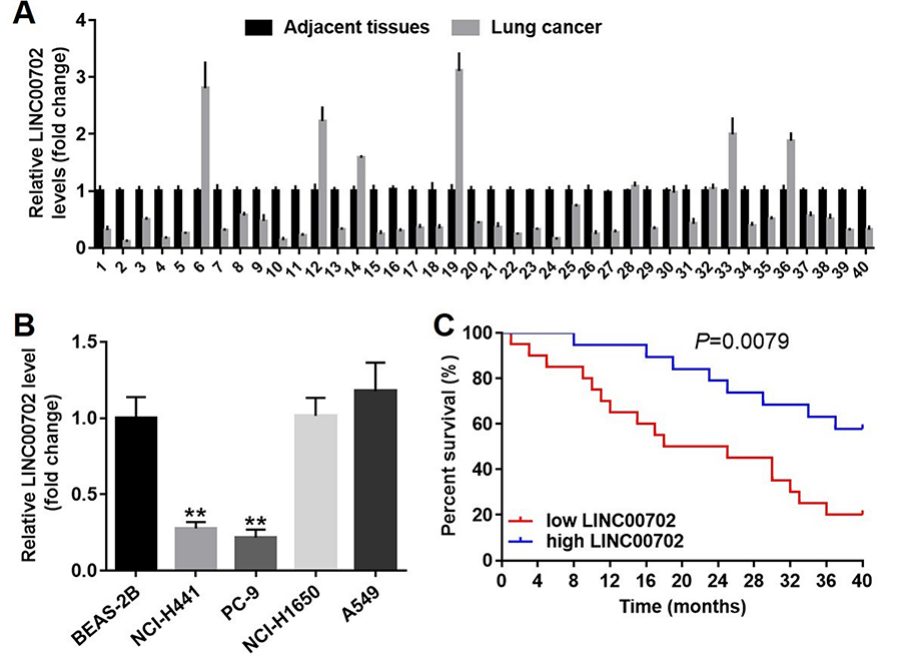
**LINC00702 was significantly downregulated in the patients with NSCLC.** (**A**) Quantitative RT-PCR analysis of relative LINC00702 expression levels in 40 pairs NSCLC tumor and adjacent tissues. (**B**) Quantitative RT-PCR analysis of relative LINC00702 expression levels in NSCLC cell lines. (**C**) LINC00702 expression was negatively associated with the survival rate of patients with NSCLC. *P<0.05, **P<0.01 was considered to indicate a statistically significant difference.

Next, to explore the clinical value of LINC00702 in NSCLC, forty pairs of tumor and adjacent normal tissues were collected from NSCLC patients. And the relative expression of the lncRNA was determined by qRT-PCR. The results indicated LINC00702 was down-regulated in 75% (30/40) NSCLC tissues compared to the adjacent normal tissues (Figure 2A). In addition, the relationship between LINC00702 expression and clinico-pathological parameters of the patients are shown in Table 1. Low levels of LINC00702 was significantly associated with increased tumor size (P<0.01), lymph node metastasis (P<0.05) and distant metastasis (P<0.05). Moreover, LINC00702 was also significantly down-regulated in the NSCLC cell lines NCI-H441 and PC-9, compared to BEAS-2B (P<0.01) (Figure 2B). Furthermore, the survival analysis demonstrated that patients with low expression of LINC00702 correlated with low overall survival (Figure 2C). Additionally, the outcome of spearman correlation test and multivariate cox regression analysis confirmed the level of LINC00702 was negatively correlated with tumor size, lymph node metastasis and distant metastasis in patients with NSCLC (Table 2 and Table 3).

**Table 1:** LINC00702 expressions correlate with clinic-pathological parameters of patients with NSCLC.

**Parameters**	**Number**	**LINC00702**	***P* value**
**Smoking status**			0.414
No	18	0.515 ± 0.613	
Yes	22	0.778 ± 0.756	
**Tumor volume**			
>4 cm	23	0.221 ± 0.213	0.005**
<4 cm	17	0.978 ± 0.844	
**Histological grade**			0.564
Poor differentiation	21	0.612 ± 0.772	
Well-intermediately differentiation	19	0.681 ± 0.672	
**Lymph node metastasis**			0.023*
N1-N3	21	0.378 ± 0.651	
N0	19	0.965 ± 0.876	
**Distant metastasis**			0.046*
M1	22	0.321 ± 0.682	
M0	18	0.789 ± 0.578	
**TNM stage**			0.334
III- IV	15	0.586 ± 0.661	
0-II	25	0.710 ± 0.789	

Student’s t test, *P<0.05, **P<0.01.

**Table 2:** Correlation between the expression of LINC00702 and clinic-pathological parameters of patients with NSCLC.

**Parameters**	**Number**	**LINC00702**	***P* value**		***r value***
**Smoking status**			0.1331		-0.2416
No	18	0.515 ± 0.613			
Yes	22	0.778 ± 0.756			
**Tumor volume**					
>4 cm	23	0.221 ± 0.213	0.0012**		-0.4951
<4 cm	17	0.978 ± 0.844			
**Histological grade**			0.3931		-0.1388
Poor differentiation	21	0.612 ± 0.772			
Well-intermediately differentiation	19	0.681 ± 0.672			
**Lymph node metastasis**			0.0026**		-0.4641
N1-N3	21	0.378 ± 0.651			
N0	19	0.965 ± 0.876			
**Distant metastasis**			0.0084**		-0.4114
M1	22	0.321 ± 0.682			
M0	18	0.789 ± 0.578			
**TNM stage**			0.2234		-0.1968
III- IV	15	0.586 ± 0.661			
0-II	25	0.710 ± 0.789			

**Table 3:** Multivariate cox regression analysis of prognosis of patients with NSCLC.

**Parameters**			**OR (95%CI)**	***P* value**
**LINC00702**			0.277 (0.087-0.888)	0.031
**Smoking status**			0.352 (0.022-5.656)	0.461
**Tumor volume**			7.111 (1.204-21.999)	0.030
**Histological grade**			0.494 (0.019-12.758)	0.671
**Lymph node metastasis**			0.486 (0.079-2.999)	0.437
**Distant metastasis**			12.110 (1.519-96.541)	0.019
**TNM stage**			0.708 (0.068-7.406)	0.773

### Overexpression of LINC00702 inhibited NSCLC cell proliferation and invasion via inducing apoptosis *in vitro*

**Figure 3:**
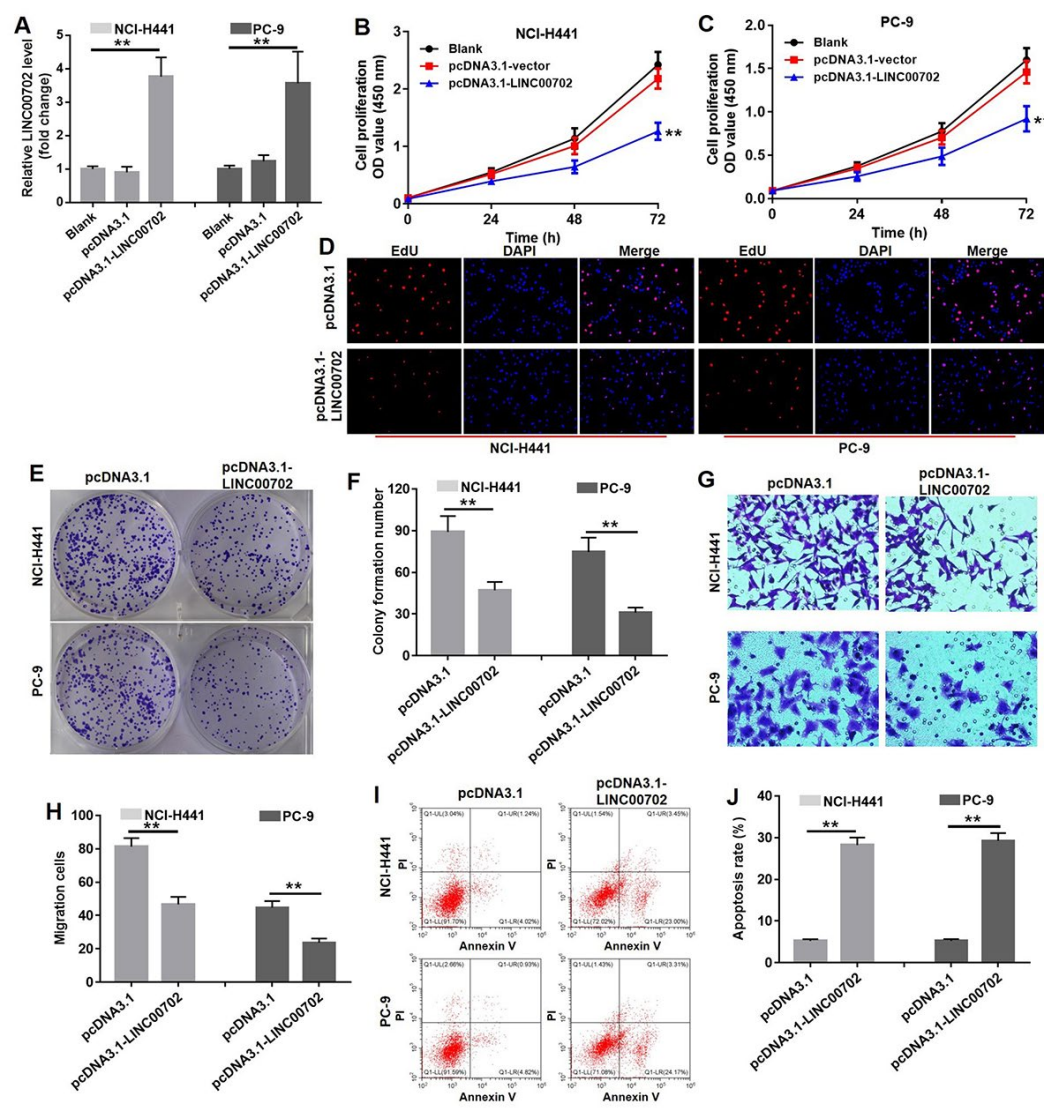
**Overexpression of LINC00702 inhibited NSCLC cell proliferation and invasion via inducing apoptosis *in vitro*.** (**A**) Quantitative RT-PCR analysis of relative LINC00702 expression levels in NCI-H441 and PC-9 transfected with pcDNA3.1, pcDNA3.1-LINC00702 or NC. (**B, C**) CCK-8 assays were used to evaluate the effect of LINC00702 on proliferation ability of NCI-H441 and PC-9 cells. NCI-H441 and PC-9 cells were transfected with pcDNA3.1 or pcDNA3.1-LINC00702 for 72 h and subjected to CCK-8 assays (**D**) EdU staining was performed in NCI-H441 and PC-9 cells. (**E, F**) Colony formation assays were used to evaluate the effect of LINC00702 on anchor-independent growth ability of NCI-H441 and PC-9 cells. NCI-H441 and PC-9 cells were transfected with pcDNA3.1 or pcDNA3.1-LINC00702 for 72 h and subjected to colony formation assays. (**G, H**). NCI-H441 and PC-9 cells were transfected with pcDNA3.1 or pcDNA3.1-LINC00702 for 72 h and subjected to transwell assay. (**I, J**) Flow cytometry was used to evaluate the effect of LINC00702 on apoptosis of NCI-H441 and PC-9 cells. **P<0.01 was considered to indicate a statistically significant difference.

To further determine the biological role of LINC00702 in NSCLC, pcDNA3.1 and pcDNA3.1-LINC00702 was transfected into NCI-H441 and PC-9 cells, respectively. As shown in Figure 3A, the levels of LINC00702 in the NCI-H441 and PC-9 cells were notably up-regulated by pcDNA3.1-LINC00702 (P<0.01), while they were not affected by pcDNA3.1 (empty vector). The result of CCK8 indicated overexpression of LINC00702 significantly decreased the proliferation of NCI-H441 and PC-9 cells by 50% and 44%, respectively (P<0.01) (Figure 3B and 3C). In addition, Edu staining further confirmed the anti-proliferation effect of LINC00702 on NSCLC cells (Figure 3D). Similarly, overexpression of LINC00702 markedly decreased the capacity of colony forming in NCI-H441 and PC-9 cells by 50% and 62%, respectively (P<0.01) (Figure 3E and 3F). Additionally, the data of transwell assay showed that overexpression of LINC00702 could significantly attenuated the invasion ability of NSCLC cells (P <0.01) (Figure 3G and 3H). Moreover, overexpression of LINC00702 dramatically induced apoptosis in NSCLC cells (P<0.01) (Figure 3I and 3J). Taken together, these results illustrated that overexpression of LINC00702 inhibited proliferation and invasion in NSCLC cells via inducing apoptosis *in vitro*.

### LINC00702 acted as a ceRNA for miR-510 in NSCLC cells

**Figure 4:**
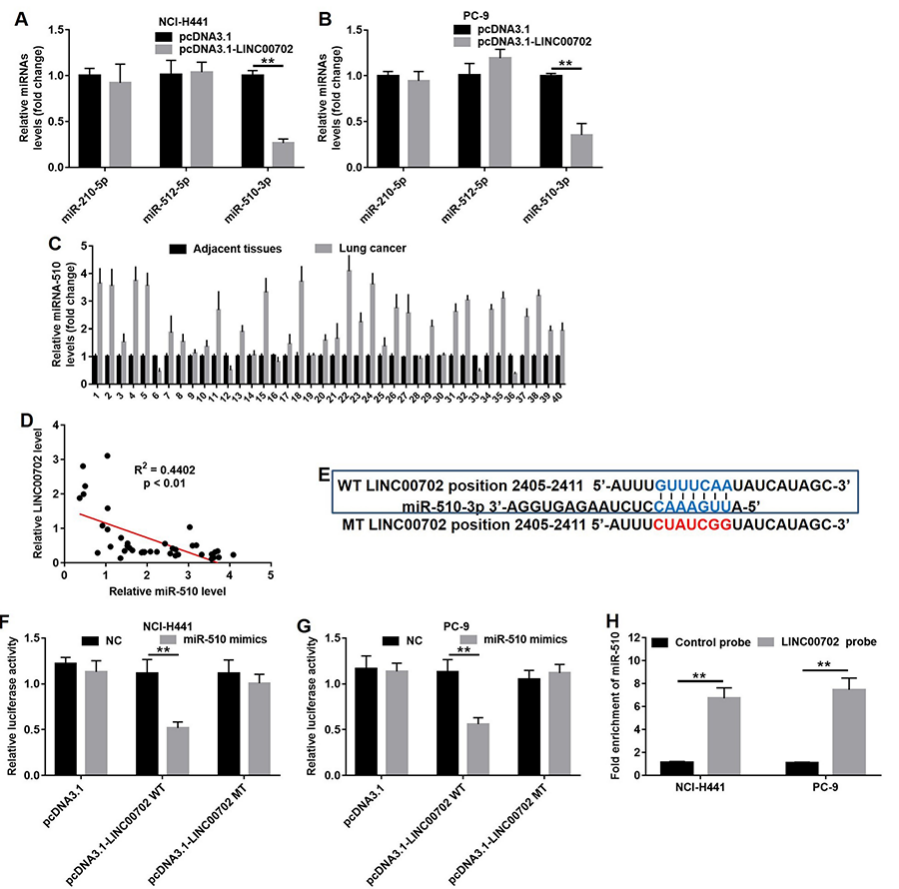
**LINC00702 functioned as a ceRNA for miR-510 in NSCLC cells.** (**A**) Quantitative RT-PCR analysis of relative miRNAs expression levels in NCI-H441 cells transfected with pcDNA3.1 or pcDNA3.1-LINC00702. (**B**) Quantitative RT-PCR analysis of relative miRNAs expression levels in PC-9 cells transfected with pcDNA3.1 or pcDNA3.1-LINC00702. (**C**) Quantitative RT-PCR analysis of relative miRNA-510 expression levels in 40 pairs NSCLC tumor and adjacent tissues. P<0.01, two-way ANOVA. (**D**) Pearson's correlation scatter plot of the fold change of LINC00702 and miR-510 in NSCLC tumor tissues. (**E**) The predicted binding sites in LINC00702 and miR-510 and the mutant sequence of LINC00702. (**F, G**) A firefly luciferase reporter containing either wild-type (WT) or mutant (MT) LINC00702 was transfected with miR-510 mimics in NCI-H441 or PC-9 cells. (**H**) LINC00702 pulled down by biotinylated miR-510 in the NSCLC cells was detected by qRT-PCR. **P<0.01 was considered to indicate a statistically significant difference.

LncRNAs often act as ceRNA by binding to miRNAs and prevent them from accessing their target mRNAs. Bioinformatics analysis (miRDB program and starBase v2.0) helped us to predict the target miRNAs regulated by LINC00702. As shown in Figures 4A and 4B, three putative target miRNAs including miR-210, miR-512 and miR-510 were identified. However, only miR-510 expression was significantly decreased in NCI-H441 (75% decrease, P<0.01) and PC-9 cells (70% decrease, P<0.01) (Figure 4A and 4B). In addition, analysis of NSCLC tumor and adjacent normal tissues showed miR-510 was upregulated in 80% (32/40) NSCLC tissues compared to their adjacent normal tissues (Figure 4C, P<0.01, two-way ANOVA). As predicted, the miR-510 expression level was negatively correlated to that of LINC00702 (R^2^=0.4402, *p*<0.01) (Figure 4D). Since the effects of miR-510 on lncRNAs are dependent on the regulation of the latter’s 3’UTR, we cloned LINC00702 with wild-type (psiCHECK2-LINC00702-WT) and mutant (psiCHECK2-LINC00702-MT) miR-510 binding sites along with luciferase reporter gene (Figure 4E). The data indicated transfection with miR-510 mimics resulted in 58% and 51% luciferase activity decrease in the NCI-H441 and PC-9 cells, respectively (p < 0.01 for both). However, the inhibitory effects were absent when the empty vector or psiCHECK2-LINC00702-MT was used (Figure 4F and 4G). Additionally, the results of pull-down assay indicated that LINC00702 was pulled down by miR-510 significantly (Figure 4H). Taken together, these findings demonstrated that miR-510 could directly bind to the 3’UTR of LINC00702.

### MiR-510 directly targeted PTEN gene

**Figure 5:**
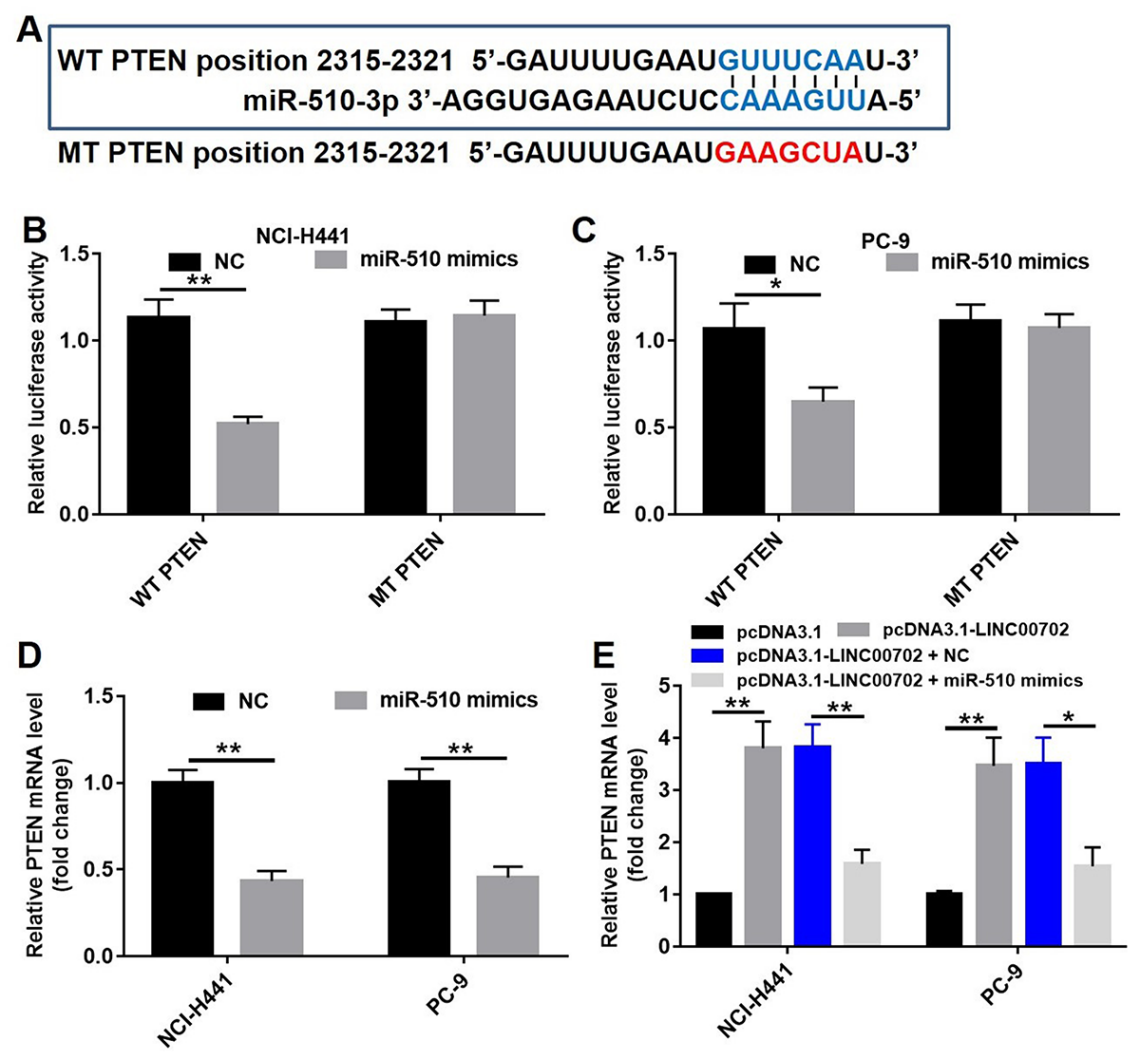
**MiR-510 directly targeted PTEN gene.** (**A**) The predicted binding sites in PTEN and miR-510 and the mutant sequence of PTEN. (**B**) A firefly luciferase reporter containing either wild-type (WT) or mutant (MT) PTEN was transfected with miR-510 mimics (20 nM) in NCI-H441 cells. (**C**) A firefly luciferase reporter containing either wild-type (WT) or mutant (MT) PTEN was transfected miR-510 mimics (20 nM) in PC-9 cells. (**D**) Quantitative RT-PCR analysis of relative PTEN expression levels in NSCLC cells transfected with control mimics or miR-510 mimics. (**E**) Quantitative RT-PCR analysis of relative PTEN expression levels in NSCLC cells transfected with pcDNA3.1-LINC00702 or/and miR-510 mimics. *P<0.05 and **P<0.01 were considered to indicate a statistically significant difference.

Previous studies have shown that PTEN, a target gene of miRNAs, acts as a tumor suppressor in NSCLC. However, the co-expression of miR-510 and PTEN in NSCLC remained unclear. Therefore, we created point mutations in the first 2-8 bases of the 3’-UTR of PTEN gene to eliminate the binding site of miR-510. The sequences of the predicted wild-type binding site of miR-510 and the corresponding mutated site are shown in Figure 5A. The wild-type (psiCHECK2-PTEN-WT) and mutant (psiCHECK2-PTEN-MT) constructs were cloned along with the luciferase reporter gene. A standard luciferase reporter assay was used to read the binding of PTEN 3’-UTR with miR-510. Compared to NC, transfection of miR-510 mimics resulted in 50% and 36% lower luciferase activity in the NCI-H441 and PC-9 cells, respectively (P<0.01) (Figure 5B and 5C). In addition, the correlation between miR-510 and PTEN expression levels was analyzed by qRT-PCR, and transfection with miR-510 significantly decreased the mRNA levels of PTEN by 60% and 55% in NCI-H441 and PC-9 cells respectively (Figure 5D, P<0.01), indicating that miR-510 could directly target PTEN.

**Figure 6:**
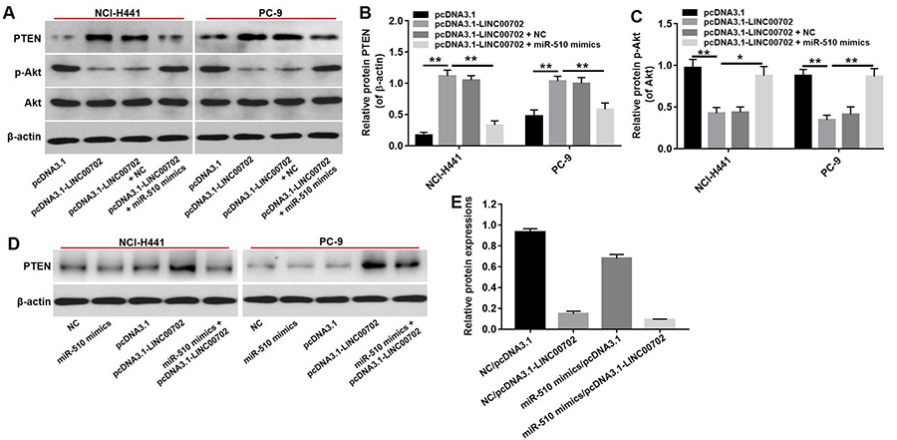
**PTEN was a target gene of miR-510.** (**A**) Western blot analysis of the expressions of PTEN, p-Akt and Akt in NSCLC cells co-transfected with pcDNA3.1-LINC00702 or/and miR-510 mimics. (**B**) Relative PTEN protein levels in NCI-H441 and PC-9 cells. (**C**) Relative p-Akt protein levels in NCI-H441 and PC-9 cells. (**D**) The expression of PTEN in NSCLC cells co-transfected with pcDNA3.1-LINC00702 or/and miR-510 mimics. (**E**) Relative PTEN expression were calculated. *P<0.05 and **P<0.01 were considered to indicate a statistically significant difference.

According to previous studies, one lncRNA could target multiple genes. Therefore, it was necessary to demonstrate that PTEN acts as a tumor suppressor through the LINC00702-miR-510 axis. As shown in Figure 5E, the PTEN mRNA levels were up-regulated by 3.9-fold and 3.8-fold respectively in pcDNA3.1-LINC00702 transfected NCI-H441 and PC-9 cells. Furthermore, the level of PTEN mRNA in cells co-transfected with pcDNA3.1-LINC00702 and miR-510 was much lower than those transfected with only pcDNA3.1-LINC00702 (Figure 5E). As expected the change of PTEN protein levels was consistent with the change of mRNA level; compared to cells transfected with the empty vector, PTEN protein levels were up-regulated by 5.5-fold and 2.5-fold respectively in NCI-H441 and PC-9 cells transfected with pcDNA3.1-LINC00702 (Figure 6A and 6B). These results demonstrated that LINC00702 regulated PTEN expression through a miR-510-dependent process.

We next investigated the effects of LINC00702 on the PTEN downstream molecule Akt. As shown in Figure 6C, p-Akt protein levels were decreased in NSCLC cells transfected with pcDNA3.1-LINC00702 compared to NC (P<0.01). However, the inhibitory effect of pcDNA3.1-LINC00702 on p-Akt expression in NSCLC cells was dramatically reversed by miR-510 mimics (Figure 6C, 6D, 6E). These results demonstrated that LINC00702 regulated PTEN pathway via directly interacting with miR-510.

### Overexpression of LINC00702 inhibited NSCLC tumor growth *in vivo*

**Figure 7:**
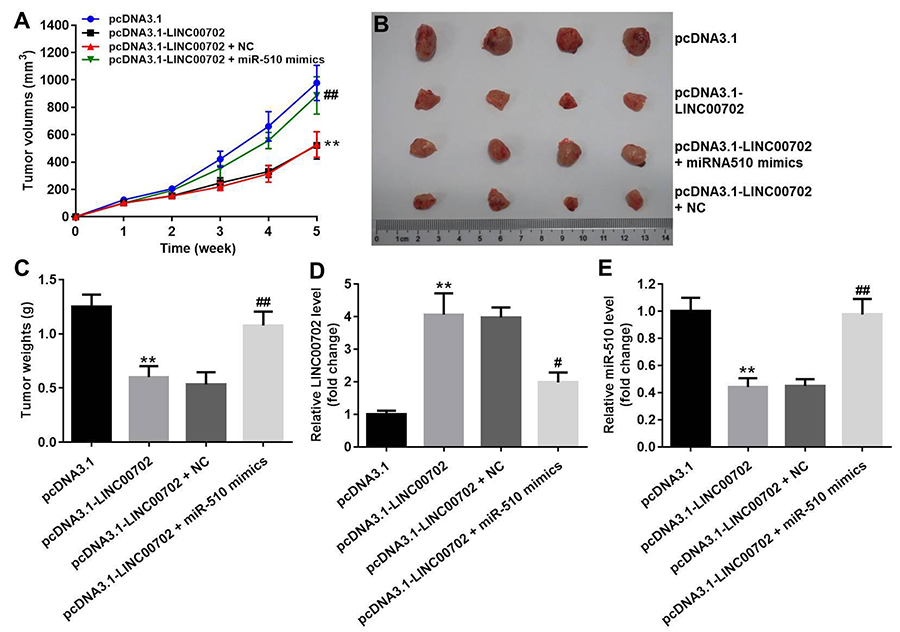
**Effects of LINC00702 on the NSCLC tumor growth *in vivo*.** (**A**) Tumor volumes in each group were monitored weekly using caliper. (**B**) Representative image of the tumors from xenografted mice. (**C**) Quantitative analysis of tumor weights isolated from mice. (**D**) Quantitative RT-PCR analysis of LINC00702 level in tumor tissues. (**E**) Quantitative RT-PCR analysis of miR-510 level in tumor tissues. **P<0.01 were considered to indicate a statistically significant difference versus the pcDNA3.1-group. ^#^P<0.05, ^##^P<0.01 were considered to indicate a statistically significant difference versus the pcDNA3.1-LINC00702 group.

**Figure 8:**
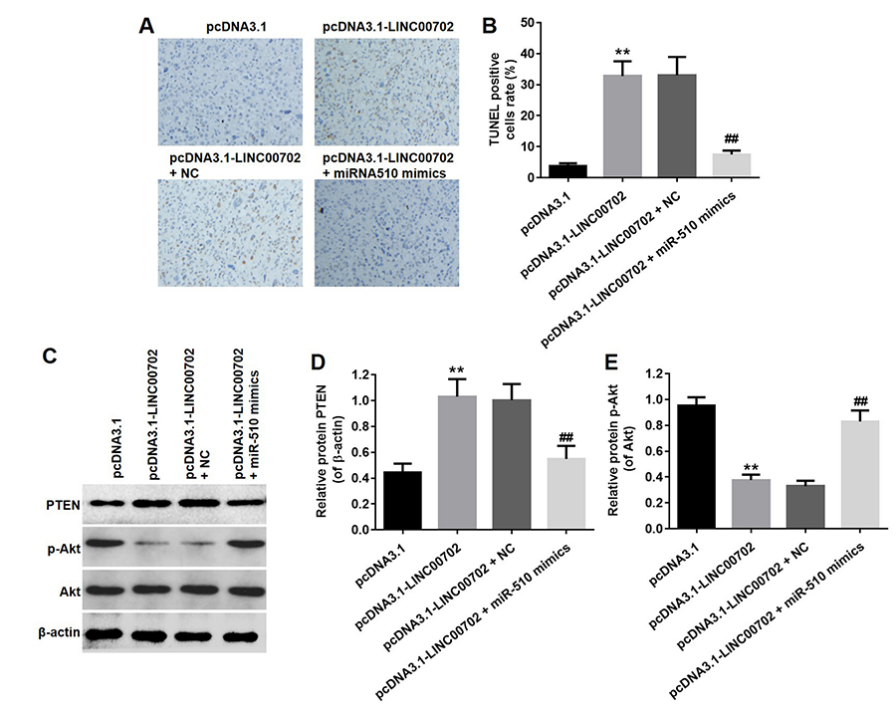
**Overexpression of LINC00702 enhanced apoptosis in tumor tissues *in vivo*.** (**A**) TUNEL analysis of apoptotic cell in the tumor tissues. (**B**) Quantification of apoptotic cells in (**A**). (**C**) Western blotting analysis of the expression levels of PTEN in tumor tissues. (**D**) Relative PTEN protein level in the experiments shown in (**C**). (**E**) Relative p-Akt protein level in the experiments shown in (**D**). **P<0.01 were considered to indicate a statistically significant difference versus the pcDNA3.1-group. ^##^P<0.01 were considered to indicate a statistically significant difference versus the pcDNA3.1-LINC00702 group.

Finally, to further validate the effects of LINC00702 and miR-510 on the growth of NSCLC *in vivo*, NCI-H441 cells transfected with pcDNA3.1-LINC00702 or/and miR-510 were subcutaneously implanted into nude mice. Consistent with *in vitro* results, NSCLC tumor growth in nude mice was significantly inhibited by pcDNA3.1-LINC00702 (Figure 7A, 7B and 7C). In addition, miR-510 mimics attenuated the anti-tumor effect of pcDNA3.1-LINC00702 on NSCLC (Figure 7A, 7B and 7C). Furthermore, the results of qRT-PCR confirmed that pcDNA3.1-LINC00702 and miR-510 mimics regulated their targets genes efficiently during the process of animal study (Figure 7D and 7E). Finally, overexpression of LINC00702 notably increased cell apoptosis in tumor tissues, which was completely reversed in the presence of miR-510 mimics (Figure 8A and 8B). Likewise, LINC00702 overexpression of significantly upregulated PTEN protein and downregulated p-Akt expression, whereas miR-510 mimics restored these effects markedly (Figure 8C, 8D and 8E). All these results further validated that LINC00702 play a tumor suppressor role in NSCLC by directly interacting with miR-510.

## DISCUSSION

Lung cancer is one of the most common malignancies in the world, and has the highest incidence among all cancers. NSCLC accounts for about 80% of all lung cancers, and its 5-year survival rate is less than 15% [1]. Since patients are usually diagnosed in the advanced stages of the disease, the only treatment available is pneumonectomy and platinum-based chemotherapy regimens. In addition, limited biomarkers, insensitive diagnostic techniques, and lack of chemotherapeutic options contribute to the suboptimal clinical outcomes in most patients with advanced NSCLC [1]. Therefore, it is important is to find novel biomarkers for effective early diagnosis, proper prognosis, and therapy of NSCLC, in addition to improving our understanding of the underlying mechanisms of NSCLC cell invasion and metastasis [13].

In this context, the non-coding RNA (ncRNA) have gained a lot of attention as potential diagnostic and therapeutic markers in NSCLC, since they are involved in tumor initiation, progression and chemosensitivity [14]. Based on their regulatory effect on tumor gene expression, lncRNAs are classified into onco-lncRNAs and tumor-suppressor lncRNAs. Metastasis-associated lung adenocarcinoma transcript1 (MALAT1) [15], colon cancer-associated transcript 2 (CCAT2) [16], HOX antisense intergenic RNA (HOTAIR) [17] are some of the oncogenic lncRNAs which promote the growth, migration and invasion of NSCLC cells. In addition, maternally expressed genes 3 (MEG3) [18], GAS6-AS1 [19], BANCR [20] are tumor suppressor lncRNAs, and their down-regulation may promote the initiation and development of NSCLC. Therefore, the study of lnRNA dynamics provide a new way to explore the mechanisms of tumorigenesis, and a new platform for developing more effective anti-tumor drugs. In this study, we firstly found LINC00702 was downregulated in NSCLC tumor tissues and was correlated with clinical outcome and prognosis of patients with NSCLC. In addition, overexpression of LINC00702 effectively inhibited NSCLC tumor growth via inducing apoptosis *in vitro* and *in vivo*.

MicroRNAs are short-chain RNAs about 22 nucleotides long, and are important post-transcriptional regulatory factors which repress gene expression by inhibiting translation or degrading the target gene [21]. LncRNAs have binding sites for specific microRNAs and act as ceRNA by sequestering them from their target genes, and relieve the inhibitory effect of miRNA and increase target gene expression. The lncRNAs therefore act as competitive endogenous RNA (ceRNA) which forms complex ceRNA networks (ceRNETs) with miRNAs and target genes [22]. A number of studies have shown that ceRNAs play crucial roles in the development of lung cancers. For example, Liu et al. found that the 3′UTR region of lncRNA AEG-1 could function as a ceRNA by inhibiting miR-30a and directly regulating Vimentin and Snail in NSCLC, thus affecting cancer development [23]. HOXD-AS1 is highly expressed in metastatic lung cancer and may function as a ceRNA in the progression of lung cancer [24]. Our findings were consistent with these studies demonstrated that LINC00702 acted as a ceRNA for miR-510 in NSCLC.

Guo et al. found that miRNA-510 was upregulated in breast cancer and was positively correlated with the invasion, migration and colony formation ability of breast cancer cells [25]. We used a bioinformatics approach to predict the target genes for miR-510a, and putatively identified PTEN, a tumor suppressor gene that is frequently mutated/deleted in cancers after the p53 and Rb genes [26]. PTEN regulates cell growth, proliferation, migration, and differentiation. The expression levels of PTEN protein and its downstream target p-AKT were significantly altered according to changes in miR-510 expression, indicating that miR-510 could regulate PTEN. Considering that LINC00702 is an up-stream regulator of the miR-510-PTEN axis, the down-regulation of LINC00702 would likely inhibited the expression of PTEN.

## CONCLUSION

In conclusion, we identified a novel signaling axis of LINC00702-miR-510-PTEN, which involved in the tumorgenesis of NSCLC. Down-regulation of LINC00702 was correlated with poor clinical outcomes and prognosis in patients with NSCLC. In addition, LINC00702 may act as a ceRNA for miR-510 and inhibited proliferation and invasion in NSCLC cells via activating the PTEN signaling pathway. Therefore, LINC00702 may serve as a potential target for the diagnosis and treatment of NSCLC.

## MATERIALS AND METHODS

### NSCLC tissue samples and cell lines

40 patients with pathologically confirmed NSCLC were enrolled for the study from The Affiliated Hospital of Qingdao University from Jan, 2015 to Feb, 2017. The informed written consent had been signed by all patients and the hospital had approved the study. Tumors were histologically graded according to the American Joint Committee on Cancer (AJCC, version 8) [10]. The human lung epithelial cell BEAS-2B, and lung cancer cell lines NCI-H441, PC-9, NCI-H1650, A549 were purchased from ATCC. All cell lines were cultured in DMEM supplemented with 10% FBS at 37°C under 5% CO_2_.

### RNA sequencing (RNA-seq) and quantitative RT-PCR (qRT-PCR) analysis

Total RNA was extracted using RNeasy96 Universal tissue kit (Qiagen, USA). Then, they were reversed to cDNA by using SuperScript III reverse transcriptase (Invitrogen) according to the manufacturer’s instructions. A TruSeq RNA library preparation kit (Illumina, USA) was used to construct Illumina-compatible libraries, and RNA-Sequencing was performed using a HiSeqTM2000 platform (Illumina, USA). Differential expressed genes (DEGs) in NSCLC and NC group was analyzed by using the edge-R package (http://www.bioconductor.org/packages/release/bioc/html/edgeR.html). In this case, the P-value was calculated according to empirical Bayesian distribution of linear models. The DEGs between NSCLC and NC were screened by the following criteria: |the log-fold change (log2FC)| ≥ 4 and false discovery rate (FDR)<0.01.

Quantitative RT-PCR was performed with PrimeScript RT reagent Kit with gDNA Eraser (TaKaRa, Japan) and SYBR Green qPCR Master Mix (TaKaRa, Japan). GADPH and U6 were used as internal controls for LINC00702 and miR-510, respectively [11]. The 2−∆∆CT method was used to measure the relative expression levels. Primers were provided by Sangon Biotech (Shanghai, China). GAPDH: forward, 5’ – ATGGCCTTCCGTGTTCCTAC-3’; reverse 5’ – CTTTACAAAGTTGTCGTTGA-3’. U6: forward, 5’-GCTTCGGCAGCACATATACTAAAAT-3’; reverse 5’-CGCTTCACGAATTTGCGTGTCAT-3’. LINC00702: forward, 5’- GCAGTGGCATGATCTCGGCT-3’; reverse 5’ -GGCCGAGGCAGGTGGATAAC-3’.

### Cell proliferation, apoptosis and colony formation assay

Cell Counting Kit-8 (CCK-8) (Sigma, USA) was used to measure cell proliferation according to the manufacturer’s instructions. Briefly, NSCLC cells (5×10^3^ cells/well) were plated into 96-well plate at 37˚ overnight, then the cells were transfected with pcDNA3.1 or pcDNA3.1-LINC00702 for 72 h. Later on, 10 μL of CKK-8 reagent was added to each well for another 1 h. The optical density (OD) value at 450 nm was detected with a microplate reader.

As for apoptosis detection, cells were washed with PBS after 72 h incubation. Then, cell suspension was incubated with 5 μL Annexin V-FITC and 5 μL propidium (PI) for 15 min at room temperature in the dark. Later on, flow-cytometer (BD, USA) was used to detect the apoptotic cells. Colony formation analysis were carried out according to previously published protocols [12].

### EdU staining

NCI-H441 and PC-9 cells were transfected with pcDNA3.1 or pcDNA3.1-LINC00702 for 72 h, then cells were subjected to EdU Alexa Fluor™ 555 staining (Thermo Fisher Scientific, USA) according to the protocol of manufacture.

### Cell invasion assay

Transwell invasion assay (Corning New York, NY, USA) was used to measure cell invasion. Briefly, the cells were seeded into 12-well plates at the density of 1×10^6^ cells per well, and serum-free DMEM was used in the upper chamber and 10% FBS supplemented DMEM in the lower chamber. The trans-well membranes were pre-coated with Matrigel (BD, Franklin Lake, NJ, USA). The cells were incubated under 5% CO_2_ at 37°C for 72 h, fixed with 70% ethanol for 10 min and stained with 0.5% crystal violet. The migrated cells were counted with an inverted microscope.

### Protein extraction and Western blotting

Total protein was extracted with RIPA buffer and separated through SDS-PAGE as per standard protocols. The proteins were then transferred to a PVDF membrane (Millipore, Billerica, MA, USA) and incubated overnight with the primary antibodies at 4°C. The membranes were incubated with the HRP-conjugated goat anti-mouse or rabbit secondary antibody, and the protein bands were visualized by ECL assay (GE Healthcare, Buckinghamshire, UK). Primary antibodies PTEN, p-Akt (Ser473), Akt and secondary antibody were provided by Cell signaling (USA).

### Plasmid construction and luciferase assay

LINC00702 was cloned into the pcDNA3.1 vector, which was transfected into NCI-H441 and PC-9 cells according to the protocol of manufacture (Genepharma, China). Then, stable NCI-H441 and PC9 cell lines expressed LINC00702 were selected by using 500 μg/ml neomycin. MiR-510 mimics or NC control were provided by Genepharma (China). Bioinformatics miRDB program (http://mirdb.org/miRDB/) was used to predict the target miRNAs regulated by LINC00702. The miR-510 binding sites in LINC00702 and PTEN were predicted by RNAhybrid 2.2 (http://bibiserv.techfak.uni-bielefeld.de/rnahybrid/). The wild-type LINC00702 (predicted binding site in 3’-UTR: 5’-AUUUGUUUCAATATCATAGC-3’), mutant LINC00702 (mutations at predicted miR-510 binding sites: 5’-ATTTCTATCGGTATCATAGC-3’), wide-type PTEN (predicted binding site in 3’-UTR: 5’-GAUUUUGAAUGUUUCAAU-3’), and mutant PTEN (mutations at predicted miR-510 binding sites: 5’-GAUUUUGAAUGAAGCUAU-3’) were synthesized by Genepharma (China). The psiCHECK2 vector was used to construct the psiCHECK2-Wt or psiCHECK2-Mut, which were transfected into NCI-H441 or PC-9 cells. A Dual-Luciferase Reporter Assay kit (Promega, USA) was used to measure the luciferase activity according to the manufacturer’s protocol.

### Pull-down assay

NCI-H441 and PC-9 cells were transiently transfected with biotinylated negative control, miR-510 or miR-510 MT (Genepharma, China) for 72 h. The cells were harvested after transfection and 50 μL of the samples were aliquoted for input. Dynabeads M-280 Streptavidin (Thermo Fisher Scientific, USA) was used for pull-down assay according to the manufacturer’s protocol. Briefly, the washed beads were incubated with equal volume of biotinylated negative control, miR-510 or miR-510 MT at 25°C in binding and washing buffer for 10 min. Later on, the immobilized miR-510 fragment enriched in the beads were incubated with 10 mM EDTA (pH = 8.2) with 95% formamide at 60°C for 10 min. Finally, RNA complexes enriched in these beads were purified using Trizol and analyzed by the qRT-PCR.

### Animal studies

Six-week-old athymic BALB/c female nude mice were obtained from the Model Animal Research Centre of Nanjing University (Nanjing, China). All animal experiments were approved by the Animal Care Committee of The Affiliated Hospital of Qingdao University. The mice were randomized into 4 groups (4 mice/group) and injected subcutaneously with 1×10^7^ NCI-H441 cells that had been transfected with pcDNA3.1-NC or pcDNA-LINC00702. 2 group of mice were intra-tumor administrated with 50 nM miR-510 mimics twice a week. The body weights of mice and tumor volumes were measured weekly. 5 weeks later, the mice were sacrificed. The tumors were resected and processed for Western blotting, qRT-PCR analyses and TUNEL staining, respectively.

### TUNEL staining

The tumor tissues were fixed in 4% formaldehyde and paraffin-embedded. In order to inhibit the endogenous peroxidase of pieces, 0.5% H_2_O_2_ was used. Then, deparaffinized tissue pieces were stained using APO-BrdU™ TUNEL Assay Kit (Thermo Fisher Scientific, Waltham, MA, USA).

### Statistical analysis

All experiments were repeated three times. Data are presented as the mean ± SD. Student’s t-test was used to compare different groups; the comparisons among multiple groups were made with one-way analysis of variance (ANOVA) followed by Dunnett’s test and two-way ANOVA. As for the survival analysis, a Kaplan Meyer Univariate test was performed. Spearman correlation test was conducted according to the marker expression and clinic-pathological variables. Multivariate cox regression was used to analyze the prognosis of patients with NSCLC. P<0.05 was considered as statistically significant.

### Ethics approval and consent to participate

Ethics approval for the study was given by The Affiliated Hospital of Qingdao University animal experimental ethics committee (201706088). National Institutes of Health guide for the care and use of laboratory animals was strictly followed by the authors.

### Consent for publication

All patients were agreeing to the publication and a written consent was obtained.

### Availability of data and materials

The datasets used and/or analyzed during the current study are available from the corresponding author on reasonable request.
